# A prediction model for hemolysis, elevated liver enzymes and low platelets syndrome in pre‐eclampsia with severe features

**DOI:** 10.1002/ijgo.15848

**Published:** 2024-08-09

**Authors:** Itamar Gilboa, Daniel Gabbai, Yariv Yogev, Omri Dominsky, Yuval Berger, Michael Kupferminc, Liran Hiersch, Eli Rimon

**Affiliations:** ^1^ Lis Hospital for Women’s Health Tel Aviv Sourasky Medical Center Faculty of Medicine Tel Aviv University Tel Aviv Israel

**Keywords:** HELLP syndrome, prediction model, pre‐eclampsia with severe features, risk factors

## Abstract

**Objective:**

The aim of the present study was to determine the risk factors for patients with pre‐eclampsia (PE) with severe features to develop hemolysis, elevated liver enzymes and low platelets (HELLP) syndrome and to design a prediction score model that incorporates these risk factors.

**Methods:**

A retrospective cohort study was conducted at a tertiary university‐affiliated medical center between 2011 and 2019. The study population comprised patients diagnosed with PE with severe features, divided into two groups: those with HELLP syndrome (study group) and those without (control group). A logistic regression was employed to identify independent predictors of HELLP syndrome. A predictive model for the occurrence of HELLP syndrome in the context of PE with severe features was developed using a receiver operating characteristic curve analysis.

**Results:**

Overall, 445 patients were included, of whom 69 patients were in the study group and 376 in the control group. A multivariate logistic analysis regression showed that maternal age <40 (OR = 2.28, 95% CI: 1.13–5.33, *P* = 0.045), nulliparity (OR = 2.22, 95% CI: 1.14–4.88, *P* = 0.042), mild hypertension (OR = 2.31, 95% CI: 1.54–4.82, *P* = 0.019), epigastric pain (OR = 3.41, 95% CI: 1.92–7.23, *P* < 0.001) and placental abruption (OR = 6.38, 95% CI: 1.29–35.61, *P* < 0.001) were independent risk factors for HELLP syndrome. A prediction score model reached a predictive performance with an area under the curve of 0.765 (95% CI: 0.709–0.821).

**Conclusion:**

This study identified several key risk factors for developing HELLP syndrome among patients with PE with severe features and determined that a prediction score model has the potential to aid clinicians in identifying high risk patients.

## Synopsis

This study identifies maternal age, nulliparity, mild hypertension, epigastric pain, and placental abruption as risk factors for HELLP syndrome, facilitating clinical prediction.

## 1 INTRODUCTION

Pre‐eclampsia (PE) complicates 2%–8% of pregnancies, and approximately a third of them develop severe features.^1^, ^2^, ^3^ The hemolysis, elevated liver enzymes and low platelets (HELLP) syndrome occurs in approximately seven per 1000 pregnancies and about 20% among patients with PE with severe features.^4^ While previous studies have recognized that HELLP syndrome is a complication of PE,^1^, ^5^ several studies have shown that patients with severe PE who also developed HELLP syndrome experienced higher rates of maternal and neonatal complications compared to those with severe PE alone.^6^, ^7^ Therefore, it is highly important for clinicians to be able to identify those patients with PE with severe features who are at higher risk to develop HELLP syndrome.

Previous studies have identified risk factors for HELLP syndrome in the general obstetric population.^8^, ^9^, ^10^ However, those studies did not specifically focus on patients with PE and severe features and, hence, there is lack of data regarding risk factors for developing HELLP syndrome among this highly selected population.

Thus, the aim of the current study was to identify risk factors for HELLP syndrome among patients diagnosed with PE with severe features and design a useful prediction score model.

## 2 MATERIALS AND METHODS

### 2.1 Study design and participants

This was a retrospective cohort study of all patients who gave birth after 24^0/7^ weeks of gestation in a single, tertiary university‐affiliated medical center, between the years 2011 and 2019. We conducted a search within our database to identify patients diagnosed with PE exhibiting severe features, and subsequently included in our analysis only those meeting the criteria according to the American College of Obstetrics and Gynecology (ACOG).^11^ Exclusion criteria included non‐viable fetuses prior to the diagnosis of PE, and patients with history of epilepsy, liver, renal, or cardiac disease as the clinical and laboratory abnormalities in those patients may be explained by their existing medical condition. Those with absence of essential information concerning maternal demographics, obstetric parameters, PE characteristics, maternal and neonatal outcomes, and postpartum follow‐up were also excluded. According to our departmental protocol, during the study period, patients in whom PE with severe features occurred after completed 34 weeks were delivered at the time of diagnosis. If PE with severe features occurred prior to 34 weeks of gestation, these patients were managed conservatively unless there were maternal or fetal indications for delivery.^11^

### 2.2 Definitions

PE with severe features was defined as one or more of the following: blood pressure (BP) during admission or hospitalization with systolic BP ≥ 160 mmHg or diastolic BP ≥ 110 mmHg on two occasions, with at least 4 h apart or elevated systolic BP between 140 and 159 mmHg or elevated diastolic BP between 90 and 109 mmHg on two occasions, with at least 4 h apart (defined as mild hypertension), with the presence of any of the following: thrombocytopenia (platelet count ≤100 000/μL); elevated liver enzymes (alanine aminotransferase [ALT] or aspartate aminotransferase [AST] ≥ twice the upper level); severe persistent right‐upper‐quadrant/epigastric pain unresponsive to medication; acute kidney injury (defined as elevated serum creatinine >1.1 mg/dL or doubling of serum creatinine in the absence of other renal disease), pulmonary edema or new‐onset of cerebral/visual disturbance.^11^ Proteinuria was defined as protein secretion ≥300 mg in a 24‐h urine collection or, when absent, as a protein/creatinine ratio ≥0.3.^11^

HELLP syndrome was defined by hemolysis (based on lactate dehydrogenase [LDH] >600 IU/L, and/or serum bilirubin ≥1.2 mg/dL, and/or a suggestive peripheral blood smear), elevated liver enzymes (ALT and AST ≥ twice the upper level), and thrombocytopenia (platelet count ≤100 000/μL).^5^ All three components were required to define patients as having HELLP.

New‐onset postpartum PE was defined as PE which occurred within 6 weeks postpartum, excluding patients with antepartum PE with persistent PE.^12^ Small for gestational age was defined as birth weight below the 10th percentile for gestational age using the local population's live‐born infant curves.^13^ As for placental abruption we included only patients in whom abruption occurred before presentation of HELLP syndrome.

### 2.3 Exposures

The exposure of interest was the development HELLP syndrome. We compared the demographic, laboratorial and obstetrical characteristics of patients with PE with severe features who developed HELLP syndrome (study group) and patients who developed PE with severe features without HELLP syndrome (control group).

### 2.4 Outcomes

The primary outcome was to determine risk factors for developing HELLP syndrome among patients with PE with severe features, and to develop a prediction score model.

Secondary outcomes were composite maternal and neonatal outcomes.

### 2.5 Statistical analysis

Univariate analysis techniques were employed to discern differences between groups. Continuous variables underwent either a two‐tailed unpaired student's *t*‐test or Mann–Whitney test (for non‐normally distributed variables), while categorical variables were analyzed using the chi‐square test or Fisher exact test. Significant variables from this analysis were subjected to binary logistic regression, followed by constructing a multivariable logistic regression model to assess the collective impact of independent variables, while controlling for significance levels (*P* < 0.05). From this, *β*‐coefficients were extracted to develop a risk prediction score, exploring the link between maternal and obstetrical characteristics and HELLP syndrome development in severe PE cases. Subsequently, a receiver operating characteristic (ROC) curve analysis was conducted to establish cutoff values, sensitivity, and specificity for predicting HELLP risk. Model discrimination, risk factors, and scores were assessed using the area under the ROC curve (AUC). Each predictor in the final model was assigned a point value based on its odds ratio (OR), which were then summed to create a risk score. The points were computed as the nearest rounded whole integer of the selected predictors' OR, then divided by 2. Finally, model performance was evaluated using ROC curves, with AUC and 95% confidence interval calculated, and prognostic accuracy assessed against a null hypothesis (area = 0.5) using a *P* value. All statistical analyses were performed using SPSS software (SPSS version 29, IBM, Chicago).

The study was approved by the local Institutional Review Board (Tel‐Aviv Sourasky Medical Center IRB, protocol number 0284‐0‐TLV, date: June 2023).

Data analysis was performed with an unidentified database. Hence, informed consent was not required.

## 3 RESULTS

Overall, 445 patients were included in the study, of whom 69 patients (15.5%) in the study group and 376 (84.5%) in the control group. The patients in the study group were younger (32.7 years vs 34.5 years; *P* = 0.047) and exhibited a higher rate of maternal age of <40 years (42.0% vs 25.3%, *P* = 0.004). In addition, they had higher rates of nulliparity (76.8% vs 63.5%, *P* = 0.033), mild hypertension (49.3% vs 22.3%, *P* < 0.001) and epigastric pain (53.6% vs 20.5%, *P* < 0.001) (Table 1).

**TABLE 1:** Maternal demographics and obstetric characteristics in the study and control groups.

	PE with severe features and HELLP (study group)	PE with severe features without HELLP	*P* value
	*n* = 69	*n* = 376 (control group)	
Maternal age at delivery (years), median (IQR)	32.7 (29.9–35.5)	34.5 (30.9–41.9)	0.042
Age <35 years, *n* (%)	46 (66.7%)	206 (54.8%)	0.067
Age <40 years, *n* (%)	56 (81.2%)	247 (65.7%)	0.011
Age <45 years, *n* (%)	62 (89.9%)	329 (87.5%)	0.582
Nulliparity, *n* (%)	53 (76.8%)	239 (63.5%)	0.033
Conception using IVF, *n* (%)	19 (27.5%)	120 (31.9%)	0.47
Pre‐gestational BMI (kg/m^2^), median (IQR)	22.9 (20.3–25.4)	23.4 (20.8–26.7)	0.312
Gestational weight gain, median (IQR)	10.0 (7.0–14.0)	12.0 (9.0–16.0)	0.014
Chronic hypertension, *n* (%)	2 (2.9%)	44 (11.7%)	0.004
Pre‐gestational diabetes, *n* (%)	1 (1.4%)	11 (2.9%)	0.487
SLE and APS, *n* (%)	2 (2.9%)	10 (4.6%)	0.457
Smoking, *n* (%)	2 (2.9%)	10 (2.7%)	0.91
Multiple gestation, *n* (%)	12 (17.3%)	50 (13.3%)	0.366
Aspirin use, *n* (%)	5 (7.2%)	48 (12.8%)	0.193
Proteinuria^a^, *n* (%)	59 (85.5%)	342 (91.0%)	0.163
Proteinuria >1 (g), *n* (%)	40 (58.0%)	215 (57.2%)	0.903
Proteinuria >5 (g), *n* (%)	13 (18.8%)	89 (23.7%)	0.308
Mild hypertension^b^, *n* (%)	34 (49.3%)	84 (22.3%)	<0.001
Systolic blood pressure (mmHg), median (IQR)	160 (144–173)	170 (160–180)	<0.001
Diastolic blood pressure (mmHg), median (IQR)	90 (86–100)	99 (90–105)	0.003
Mean arterial pressure (mmHg), median (IQR)	113 (107–123)	122 (112–130)	<0.001
Headache, *n* (%)	15 (21.7%)	97 (25.8)	0.475
Epigastric pain, *n* (%)	37 (53.6%)	77 (20.5%)	<0.001
Visual changes, *n* (%)	5 (7.2%)	57 (15.2%)	0.081
Corticosteroid administration, *n* (%)	28 (40.6%)	113 (30.1%)	0.084

Abbreviations: APS, anti‐phospholipid syndrome; BMI, body mass index; HELLP, hemolysis, elevated liver enzymes and low platelets; IQR, interquartile range; IVF, in vitro fertilization; SLE, systemic lupus erythematous; SD standard deviation.

^a^ Proteinuria is defined as microalbumin ≥300 mg in a 24‐h urine collection or, when absent, as a protein/creatinine ratio ≥0.3 mg/mmol.

^b^ Elevated systolic blood pressure between 140 and 159 mmHg or elevated diastolic blood pressure between 90 and 109 mmHg on two occasions, with at least 4 h apart.

Patients in the study group had higher rates of preterm birth <34 weeks of gestation (42.0% vs 25.3%, *P* = 0.004) but not preterm birth <37 weeks of gestation (71.0% vs 59.3%, *P* = 0.067) as compared to the control group. In addition, there was higher rate of placental abruption in the study group (5.8% vs 1.3%, *P* = 0.015) (Table 2).

**TABLE 2:** Maternal outcomes in the study and control groups.

	PE with severe features and HELLP	PE with severe features without HELLP	*P* value
	*n* = 69	*n* = 376	
Delivery <34 weeks, *n* (%)	29 (42.0%)	95 (25.3%)	0.004
Maternal indication, *n* (%)	27 (93.1%)	72 (75.8%)	0.042
Fetal indication, *n* (%)	2 (6.9%)	20 (21.1%)	0.081
Delivery <37 weeks, *n* (%)	49 (71.0%)	223 (59.3%)	0.067
Early PE <34 weeks, *n* (%)	30 (43.5%)	126 (33.5%)	0.111
Placental abruption, *n* (%)	4 (5.8%)	5 (1.3%)	0.015
New‐onset postpartum PE, *n* (%)	5 (7.2%)	33 (8.8%)	0.676
Gestational diabetes mellitus, *n* (%)	8 (11.6%)	50 (13.3%)	0.699
Cesarean section, *n* (%)	59 (85.5%)	300 (79.8%)	0.269
Non‐elective CS, *n* (%)	58 (84.1%)	282 (75.0%)	0.103
ICU admission, *n* (%)	5 (7.2%)	19 (5.1%)	0.458
Postpartum cardiomyopathy, *n* (%)	1 (1.4%)	5 (1.3%)	0.937
Dyspnea, *n* (%)	4 (5.8%)	12 (3.2%)	0.285
Acute kidney injury, *n* (%)	12 (17.4%)	48 (12.8%)	0.301
Pulmonary edema, *n* (%)	1 (1.4%)	14 (3.7%)	0.336
Eclampsia, *n* (%)	0	9 (2.4%)	0.196

Abbreviations: CS, cesarean section; HELLP, hemolysis, elevated liver enzymes and low platelets; ICU, intensive care unit; IQR, interquartile range; PE, pre‐eclampsia; SD, standard deviation.

Neonatal outcomes are presented in Table S1. There were higher rates of low 5‐min Apgar score and intraventricular hemorrhage in newborns of patients in the study group compared to the controls.

In a multivariate analysis adjusted for maternal age, chronic hypertension, nulliparity, gestational age at birth, delivery before 34 weeks' gestation, birth weight, antepartum steroids, newborn gender, severe hypertension and placental abruption, HELLP syndrome was found to be an independent risk factor for 5‐min Apgar score <7 (OR = 4.73, 95% CI: 1.62–13.31, *P* = 0.004).

Furthermore, in the multivariate analysis assessing risk factors for HELLP syndrome, the following variables were found to be independently associated with HELLP syndrome: maternal age <40 years (OR = 2.28, 95% CI: 1.13–5.33, *P* = 0.045), mild hypertension (OR = 2.31, 95% CI: 1.54–4.82, *P* = 0.019), nulliparity (OR = 2.2, 95% CI: 1.14–4.88, *P* = 0.042), epigastric pain (OR = 3.41, 95% CI: 1.92–7.23, *P* < 0.001), and placental abruption (OR = 6.38, 95% CI: 1.29–35.61, *P* < 0.001).

**FIGURE 1:**
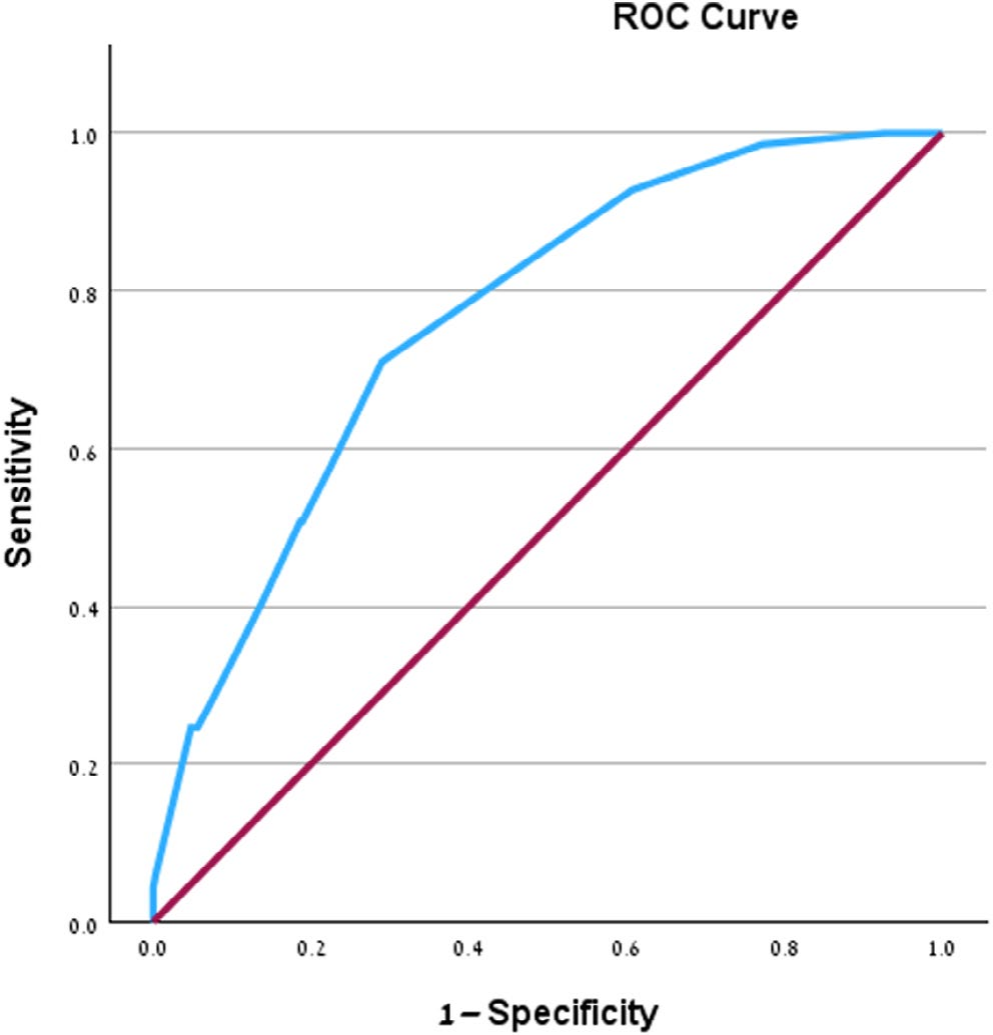
Receiver operating characteristic curve (ROC) analysis for predictive model performance. Diagonal segments are produced by ties.

We further established a scoring system based on the aforementioned multivariable logistic regression analysis to assess the risk of developing HELLP syndrome among patients with PE, as outlined in Table 3. The included predictors were assigned scores correlating with their relative weights, calculated as the sum of the predictors' weights as follows: risk score = ([1 × age < 40 years] + [1 × nulliparity] + [1 × mild hypertension] + [1.5 × epigastric pain] + [3 × placental abruption]). The total scores for each individual ranged from 0 to 7.5. As illustrated in Table 4, the scores were used to define four risk groups: A = score of 0–2, B = score of 2.5–3.5, C = score of 4–4.5, and D = score of ≥5. The risk of developing HELLP syndrome was positively correlated with higher scores. Using a ROC curve (see Figure 1), we found that the model had the ability to predict the occurrence of HELLP syndrome, as indicated by an AUC of 0.765 (95% CI: 0.709–0.821, *P* < 0.001).

**TABLE 3:** Model score for HELLP syndrome among patients with PE with severe features.

Age <40	
Yes	1
No	0
Mild hypertension	
Yes	1
No	0
Nulliparity	
Yes	1
No	0
Epigastric pain	
Yes	1.5
No	0
Placental abruption	
Yes	3
No	0

Abbreviations: HELLP, hemolysis, elevated liver enzymes and low platelets; PE, pre‐eclampsia.

**TABLE 4:** Score points and risk groups.

Score points	HELLP (risk group %)	Risk group	Calculated risk (risk group %)
0	0/27	A	8.10%
1	5/126 (4.0%)		
1.5	1/9 (11.1%)		
2	19/147 (12.9%)		
2.5	8/30 (26.6%)	B	27.60%
3	7/26 (26.9%)		
3.5	12/42 (28.6%)		
4	0/4	C	38.20%
4.5	13/30 (43.3%)		
5	1/2 (50%)	D	80%
≥5.5	3/3 (100%)		

Abbreviation: HELLP, hemolysis, elevated liver enzymes and low platelets.

## 4 DISCUSSION

In the current study, we aimed to investigate the risk factors associated with HELLP syndrome in patients with PE and severe features. Our main findings were: (1) maternal age <40 years, nulliparity, mild hypertension, epigastric pain, and placental abruption are independent risk factors for HELLP syndrome. (2) Utilizing these identified variables, we formulated a prediction score model, achieving a predictive performance with an AUC of 0.765. This model holds potential for identifying individuals at a heightened risk of developing HELLP syndrome within the context of PE with severe features.

Our findings regarding the susceptibility of patients with PE with severe features under the age of 40 to develop HELLP syndrome contrast with earlier studies which showed that advanced maternal age is associated with a higher risk of HELLP syndrome.^9^, ^10^ However, these studies investigated the general population's incidence of HELLP syndrome in relation to maternal age. Importantly, studies focusing on a specific subset of patients who had PE with severe features, with and without HELLP syndrome, found comparable maternal age in both groups, but the authors did not investigate the correlation between maternal age and development of HELLP syndrome.^6^, ^7^ In line with previous studies, we also found that nulliparity is a risk factor for developing HELLP syndrome.^8^, ^9^ This association is often explained by immune maladaptation contributing to the increased risk of PE in nulliparous patients.^14^, ^15^ Bdolah et al. suggested that it may be attributed to elevated levels of circulating soluble fms like tyrosine kinase 1 (sFlt1) to placental growth factor (PlGF) ratio among nulliparous with PE,^16^ a finding also observed in patients with HELLP syndrome.^17^

The typical presentation of HELLP syndrome involves hypertension and proteinuria. However, approximately 10%–20% of patients may not demonstrate proteinuria,^4^, ^18^ a finding consistent with our study, where 14.5% of patients did not exhibit proteinuria. Furthermore, our study revealed that patients who developed HELLP syndrome had lower mean arterial systolic pressure. This suggests that the severity of hypertension does not necessarily correlate with the incidence of maternal and neonatal complications.^19^

Our study identified epigastric pain as an independent risk factor for the development of HELLP syndrome, aligning with previous reports that have recognized epigastric pain as a prevalent symptom in patients diagnosed with HELLP syndrome, often indicating a liver involvement.^4^, ^18^, ^20^ Notably, we found that placental abruption is independently associated with HELLP syndrome in our high‐risk population of patients with PE and severe features. Consistent with our findings, Maged et al. and Vinnar et al. also showed an association between placental abruption and development of HELLP syndrome among patients with PE and severe features.^7^, ^21^ This association was previously described by others, including Sibai et al. and Audibert et al., suggesting potential shared pathophysiological mechanisms or common risk factors in both conditions.^18^, ^20^

### 4.1 Significance of the study

The primary significance of the current study lies in the development of a unique risk score model for clinicians to identify patients with PE with severe features who are at a higher risk of developing HELLP syndrome. In a prospective study, Malte et al. found that C‐terminal pro‐endothelin‐1 (CT‐pro‐ET‐1), sFlt1, and high systolic BP accurately predicted progression to severe PE/HELLP syndrome within 1–2 weeks (AUC of 0.94).^22^ However, their focus was on patients with PE without severe features and their risk of progressing to severe PE/HELLP syndrome. More recently, Li et al. developed a prediction model utilizing various biochemical markers to anticipate the progression from gestational hypertension to HELLP syndrome.^23^ Their findings indicated that several biochemical markers, such as hemoglobin and the AST to platelet ratio, were associated with an elevated risk of developing HELLP syndrome. In contrast to our study, which concentrated on patients with PE featuring severe symptoms who subsequently developed HELLP syndrome, the authors evaluated patients with gestational hypertension who progressed to mild PE and HELLP. Importantly, we could not identify a model specifically tailored to predict HELLP syndrome among patients with PE with severe features.

### 4.2 Strengths and limitations

The main strength of our study was the large cohort of highly selected patients with PE with severe features, all delivered in a single tertiary medical center. We defined PE with severe features according to new ACOG guidelines^11^ to ensure updated recommendations, and HELLP syndrome was strictly defined. On the other hand, a major limitation of this study was its retrospective design and limited data on additional prognostic markers, which could have enhanced the accuracy of our model. Furthermore, the study lacked information on various prognostic parameters such as gestational age at the diagnosis of gestational hypertension, familial history of PE, previous history of PE, and levels of various angiogenic markers. Unfortunately, data regarding the levels of sFlt1 and PlGF were unavailable for patients in this cohort, as the use of these tests in clinical practice was recently incorporated.

## 5 CONCLUSIONS

In this study, we identified distinct risk factors associated with the development of HELLP syndrome in patients with severe features of PE. Maternal age below 40 years, nulliparity, mild hypertension, epigastric pain, and placental abruption were established as independent risk factors for HELLP syndrome in this subgroup of PE patients. Subsequently, our analysis led to the formulation of a prediction score model with a high‐performance index. This model has the potential to aid clinicians in identifying patients at an elevated risk of developing HELLP syndrome. Nevertheless, it is crucial for future studies to validate these findings and refine the predictive model for enhanced accuracy.

## AUTHOR CONTRIBUTIONS

Itamar Gilboa: Conceptualization, data curation, investigation, writing—original draft, review and editing. Daniel Gabbai: Data curation, writing—review and editing. Yariv Yogev: Conceptualization, writing—validation, review and editing. Omri Dominsky: Data curation, writing—review and editing. Yuval Berger: Data curation, writing—review and editing. Michael Kupferminc: Writing—validation, review and editing. Liran Hiersch: Conceptualization, writing—validation, review and editing. Eli Rimon: Conceptualization, data curation, writing—original draft, review and editing, project administration. All authors revised the final manuscript.

## FUNDING INFORMATION

This research received no external funding.

## CONFLICT OF INTEREST STATEMENT

All authors report no conflict of interest and no financial disclosures. We confirm that there are no instances of plagiarism in our manuscript.

## Supporting information


Table S1.

## Data Availability

The data that support the findings of this study are available from the corresponding author upon reasonable request.
