# Supplementary material for: A prediction model for hemolysis, elevated liver enzymes and low platelets syndrome in pre‐eclampsia with severe features
Source: Int J Gynaecol Obstet. 2024 Aug 9;168(1):230–6. doi: 10.1002/ijgo.15848 (PMC11649887; doi:10.1002/ijgo.15848)
Supplement: Supplementary file 1 — Table S1. [file IJGO-168-230-s001.docx]

**Supplementary Table 1**: Neonatal outcomes in the study and control groups.

| P-value | PE with severe features without HELLP  n=376 | PE with severe features and HELLP  n=69 |  |
| --- | --- | --- | --- |
| 0.839 | 200 (43.8%) | 38 (42.7%) | Gender – male, n(%) |
| 0.004 | 2258.2 (±773.8) | 1988.49 (±784.9) | Birthweight (gr ), mean (±SD) |
| 0.051 | 25.5 (9.0-52.0) | 18.5 (9.0-39.0) | Birthweight percentile, median (IQR) |
| 0.364 | 111 (26.0%) | 25 (30.9%) | Birthweight < 10%, n (%) |
| 0.888 | 39 (9.1%) | 7 (8.6%) | Birthweight < 3%, n (%) |
| 0.232 | 26 (6.1%) | 4 (4.9%) | Amniotic fluid - meconium, n (%) |
| <0.001 | 23 (5.4%) | 16 (19.8%) | 5-minutes Apgar score <7, n (%) |
| 0.534 | 10 (2.4%) | 1 (1.3%) | Umbilical cord pH <7.1, n (%) |
| 0.490 | 66 (15.5%) | 15 (18.5%) | NICU, n (%) |
| 0.758 | 34.9 (±25.6) | 32.7 (±19.7) | NICU length of hospitalization (days), mean (±SD) |
| 0.157 | 54 (12.6%) | 15 (18.5%) | RDS, n (%) |
| 0.987 | 52 (12.4%) | 10 (12.3%) | Hypoglycemia, n (%) |
| 0.503 | 19 (4.4%) | 5 (6.2%) | NEC, n (%) |
| 0.040 | 12 (2.8%) | 6 (7.4%) | IVH, n (%) |
| 0.328 | 5 (1.2%) | 0 | Neonatal death, n (%) |

Abbreviations: NICU, neonatal intensive care unit; RDS, respiratory distress syndrome; NEC, necrotizing enterocolitis; IVH, intraventricular hemorrhage; SD standard deviation; IQR, interquartile range
